# Determination of MIC Quality Control Parameters for Exebacase, a Novel Lysin with Antistaphylococcal Activity

**DOI:** 10.1128/JCM.03117-20

**Published:** 2021-06-18

**Authors:** Maria M. Traczewski, Jane E. Ambler, Raymond Schuch

**Affiliations:** a The Clinical Microbiology Institute, Wilsonville, Oregon, USA; b ContraFect Corporation, Yonkers, New York, USA; Medical College of Wisconsin

**Keywords:** exebacase, CF-301, lysin, QC range, lysin

## Abstract

Exebacase (CF-301), a novel, antistaphylococcal lysin (cell wall hydrolase) is the first agent of this class to enter late-stage clinical development (phase 3, NCT04160468) for the treatment of Staphylococcus aureus bacteremia, including right-sided endocarditis. A multilaboratory Clinical and Laboratory Standards Institute (CLSI) M23-defined tier 2 quality control (QC) study was conducted to establish exebacase QC ranges for a new reference broth microdilution method. S. aureus ATCC 29213 and Enterococcus faecalis ATCC 29212 were selected as reference QC strains. Broth microdilution MIC QC ranges for exebacase spanned 4 log_2_ dilutions and contained 99.2% of the MIC results generated for the two reference strains. The QC ranges for exebacase were defined as 0.25 to 2 μg/ml and 8 to 64 μg/ml against S. aureus ATCC 29213 and E. faecalis ATCC 29212, respectively, and were approved by the CLSI Subcommittee on Antimicrobial Susceptibility Testing. These QC ranges established for use with the reference broth microdilution method developed for exebacase susceptibility testing will ensure the test performance and accuracy of results generated during clinical development.

## INTRODUCTION

Exebacase is a first-in-class protein therapeutic, organized into two distinct functional domains, a C-terminal domain, which binds with high affinity to conserved peptidoglycan ligands or cell wall-associated glycopolymers and an N-terminal catalytic domain that enzymatically cleaves peptidoglycan bonds within the cell wall. Exebacase elicits direct lytic activity against specific target bacteria and thereby demonstrates the following microbiological attributes: rapid, specific bactericidal activity; the ability to eradicate staphylococcal biofilms; synergy with antistaphylococcal antibiotics; a low propensity to develop resistance; no cross-resistance with antibiotics; and an extended postantibiotic effect (1–4).

In preliminary *in vitro* susceptibility studies, pronounced trailing was observed for all Staphylococcus aureus isolates tested in triplicate, including the quality control (QC) strains, using cation-adjusted Mueller-Hinton broth (CAMHB) (5). Trailing frequently obscured MIC endpoints and resulted in poor MIC reproducibility. Additionally, a loss of activity was reported when frozen form MIC panels (prepared with CAMHB) were used for MIC testing, yielding higher MIC results. For these reasons, modifications to the standard broth microdilution reference method were systematically investigated following protocols outlined in CLSI M23-A3 in order to eliminate the trailing effect and develop a reference broth microdilution method for testing exebacase susceptibility against clinical isolates (5, 6). A modified broth microdilution method using CAMHB supplemented with 25% horse serum and 0.5 mM dl-dithiothreitol was developed at ContraFect for this first-in-class protein-based therapeutic and approved by the CLSI Subcommittee on Antimicrobial Susceptibility Testing in 2017 (7). The development of this modified broth microdilution method and the conduct of initial M23-style QC (tier 1) studies to identify preliminary QC ranges are described elsewhere (5).

Early establishment of expected QC ranges is necessary for the effective evaluation of antibacterial test performance and to thereby ensure that accurate, reproducible, and robust MIC data are generated in clinical development. Exebacase susceptibility testing of clinical trial isolates was performed in the recently completed phase 2 (ClinicalTrials.gov identifier NCT03163446) superiority design study that established proof of concept for exebacase as a potential to treatment for methicillin-resistant S. aureus (MRSA) bloodstream infections (16). As for all new antibacterial agents, it is essential to monitor QC data continually during clinical trials and to note deviations from the modal MIC as well as potential trending of higher or lower MIC values, in particular when an unfamiliar modified method is to be employed by a central laboratory and testing large numbers of clinical isolates collected as part of a large antibacterial surveillance program. Traczewski et al. reported exebacase *in vitro* activity using the modified broth microdilution method (MIC values of ≤1 μg/ml) against 535 S. aureus (MRSA and methicillin-susceptible S. aureus [MSSA]) isolates collected from medical centers in the United States, Europe, and South America (8). The current report describes the design and results of a multilaboratory broth microdilution MIC QC study conducted to evaluate expected exebacase MIC QC ranges against relevant ATCC strains recommended by CLSI.

(Data from this study were presented at the ASM Microbe 2017, New Orleans, LA, 1 to 5 June 2017 [9].)

## MATERIALS AND METHODS

### Participating laboratories.

Eight independent laboratories (laboratory directors shown in parentheses) participated in this study by performing broth microdilution MIC testing following CLSI guidelines (M07-A10 [10] and M100 [11]): Clinical Microbiology Institute, Wilsonville, OR (Maria M. Traczewski); the UCLA Medical Center, Microbiology Laboratory, Los Angeles, CA (Janet Hindler); the University of Rochester Medical Center, Microbiology Laboratory, Rochester, NY (Dwight J. Hardy); International Health Management Associates Inc., Schaumburg, IL (Dana Dressel); Thermo Fisher Scientific, Oakwood Village, OH (Cindy Knapp); Laboratory Specialists Inc., Westlake, OH (Laura M. Koeth); Tufts New England Medical Center, Boston, MA (Laura McDermott); Micromyx, Kalamazoo, MI (Chris Pillar). The Clinical Microbiology Institute coordinated the distribution of necessary laboratory supplies to the other seven laboratories and conducted the analysis of the MIC results.

### Antibacterial agents.

Exebacase (molecular weight, 25,928 Da) was provided by ContraFect Corp., Yonkers, NY, as frozen solution (lot NB0020-11-13) at a concentration of 10 mg/ml in a buffer (pH 7) containing 20 mM l-histidine and 5% d-sorbitol. Vancomycin (lot SLBM3283V) purchased from Sigma-Aldrich, St. Louis, MO, was used as an internal control test agent. A vancomycin stock solution was prepared by dissolving in water and diluted to a final concentration of 1,280 μg/ml.

### Bacterial isolates and culture media.

The two CLSI-recommended QC strains selected for testing were S. aureus ATCC 29213 and Enterococcus faecalis ATCC 29212. Exebacase has specific activity against Staphylococcus species and group A, B, and G Streptococcus species (12). Since exebacase has no clinically relevant activity against E. faecalis, QC ranges were established against E. faecalis ATCC 29212 in order to evaluate the performance of the test method should isolates with exebacase MICs of >2 μg/ml be encountered.

The Clinical Microbiology Institute prepared three lots of broth microdilution panels to test exebacase susceptibility. Exebacase stock solution was diluted (0.12 to 128 μg/ml) using three different commercial lots of Mueller-Hinton broth (MHB) manufactured by three different companies: lot 1 was BBL Mueller-Hinton II (cation-adjusted) broth (catalog no. 212322, lot 5257869; Becton Dickinson, Franklin Lakes, NJ) and required no adjustment for cations, whereas lot 2 from Difco Mueller-Hinton Broth (catalog no. 275730, lot 4045151; Becton Dickinson) and lot 3 from Oxoid Mueller-Hinton Broth (catalog no. CM0405B, lot 1743805; Thermo Fisher Scientific, Bedford, MA) required supplementation with divalent cations Mg^2+^ and Ca^2+^ after sterilization as described in CLSI standard M07-A10 (10). All three lots were supplemented with 25% horse serum (Sigma-Aldrich) and 0.5 mM dl-dithiothreitol (Sigma-Aldrich); following the aseptic addition of these supplements, the pH ranged from 7.2 to 7.4. Vancomycin solution was diluted in lot 1 BBL Mueller-Hinton II (cation-adjusted) broth without supplementation (horse serum or dl-dithiothreitol). All freshly prepared MIC panels were placed in plastic bags and immediately frozen at −70°C, shipped to each of the participating laboratories frozen, and stored at −70°C until use.

### Broth microdilution susceptibility testing.

Quality control strains S. aureus ATCC 29213 and E. faecalis ATCC 29212 were subcultured from freezer stock 2 days prior to testing. Each laboratory tested 10 replicates of each QC strain, and the number of replicates tested per day was left to the discretion of each of the participating laboratories; however, in accordance with CLSI M23-A3 guidelines, all testing was performed within a minimum of 3 days. Each laboratory generated a total of 30 MIC results for each of the two QC strains tested (an MIC result for each of the 10 replicates, tested in three different lots of CAMHB). Inocula were prepared in 0.85% saline to match a McFarland 0.5 turbidity standard using colonies taken from a subculture on sheep blood agar plate incubated overnight for 18 to 24 h prior to each test day. Each replicate was tested using a unique 0.5 McFarland preparation. Each inoculum preparation was further diluted with saline to obtain a final inoculum of approximately 5 × 10^5^ CFU/ml in the test panel. For S. aureus ATCC 29215, the modal inoculum colony count was 4.8 × 10^5^ CFU/ml, and for E. faecalis, it was 4.4 × 10^5^ CFU/ml. The inoculated MIC panels were incubated according to CLSI M07 (10). Each laboratory performed colony counts of the inoculum density on drug-free agar, on each day of testing. Appropriate assay performance was verified by concurrent MIC testing of vancomycin as an internal control agent. Laboratories were instructed to perform repeat tests if any of the vancomycin results were out of range on the day of testing.

### Analysis of QC ranges.

All MIC and colony count data reported were submitted to the Clinical Microbiology Institute for analysis. The study results were analyzed using criteria outlined in M23-A3 (6), and analyses of MIC results were done using the RangeFinder statistical program and methods described by Turnidge and Bordash (13).

## RESULTS

The exebacase broth microdilution MIC results (in micrograms per milliliter) against S. aureus ATCC 29213 and E. faecalis ATCC 29212 for each participating laboratory and each of the three commercial MHB lots tested are presented in Table 1.

**TABLE 1:** Medium lot and inter- and intralaboratory comparisons of exebacase broth microdilution MICs obtained against S. aureus ATCC 29213 and E. faecalis ATCC 29212

Strain and exebacase MIC (μg/ml)	No. of occurrences for CAMHB lot^*a*^:			No. of occurrences at laboratory:								Total/mean/ range
	1	2	3	1	2	3	4	5	6	7	8	
**ATCC 29213**^*b*^												
0.25	17						10		5		2	17
0.5	63	38	6	18	12	10	10	16	17	12	12	107
1		41	61	12	16	11	10	12	8	17	16	102
2		1	11		1	8		2		1		12
4			2		1	1						2
**Total**	**80**	**80**	**80**	**30**	**30**	**30**	**30**	**30**	**30**	**30**	**30**	**240**
Geo mean	0.43	0.73	1.08	0.66	0.81	1	0.5	0.72	0.54	0.78	0.69	0.70
Log_2_ range	2	3	4	2	4	4	3	3	3	3	3	5
**ATCC 29212**^*c*^												
8	4		3				1	6				7
16	73	13	47	17	12	11	25	13	20	16	19	133
32	3	58	26	13	11	16	4	11	9	13	10	87
64		8	3		5	3			1	1	1	11
128		1	1		2							2
**Total**	**80**	**80**	**80**	**30**	**30**	**30**	**30**	**30**	**30**	**30**	**30**	**240**
Geo mean^*d*^	15.86	31.18	21.11	21.61	29.86	26.60	17.15	17.96	20.63	22.63	21.11	21.86
Log_2_ range	3	4	5	2	4	3	3	3	3	3	3	5

a Lot 1 = BBL Mueller-Hinton II (cation-adjusted) broth; lot 2 = Difco Mueller-Hinton broth; lot 3 = Oxoid Mueller-Hinton broth.

b The gray-shaded box represents the exebacase quality control range of four dilutions (0.25 to 2 μg/ml) for ATCC 29213 (99.2% included, 238/240).

c The gray-shaded box represents the exebacase quality control range of four dilutions (8 to 64 μg/ml) for ATCC 29212 (99.2% included, 238/240).

d Geometric mean of observed values.

For S. aureus ATCC 29213, the overall distribution of MIC results was 5 log_2_ dilution steps (0.25 to 4 μg/ml), 44.6% of results were at the mode (0.5 μg/ml), and 94.2% of results were within 1 doubling dilution of the mode. The MIC range for each laboratory was 2 to 4 log_2_ dilution steps. The results were analyzed by individual medium lots: for commercial MHB lots 2 and 3, which required supplemental cation adjustments (M07), the distribution of MIC values was 3 and 4 log_2_ dilutions, with 51.3% and 76.2% of results at the mode (1 μg/ml) and 100% and 97.2% of results within 1 doubling dilution of the mode, respectively. However, MHB lot 1, which required no cation adjustment, generated an MIC distribution of 2 log_2_ dilutions, with 78.7% of results at the mode (0.5 μg/ml) and 100% within 1 doubling dilution of the mode.

For E. faecalis ATCC 29212, the overall distribution of MIC results was 5 log_2_ dilution steps (8 to 128 μg/ml), 55.4% of results were at the mode (16 μg/ml) and 94.6% of results were within 1 doubling dilution of the mode. The MIC range generated by each laboratory was 2 to 4 log_2_ dilution steps. When analyzed by individual medium lots, with commercial MHB lots 2 and 3, the distributions of MIC values were 4 and 5 log_2_ dilutions, with 72.5% and 58.7% of results at the mode (32 and 16 μg/ml, respectively) and 100% and 97.2% of results within 1 doubling dilution of the respective modes for each lot. Whereas less variability in MIC results was reported with MHB lot 1 which generated an MIC distribution of 3 log_2_ dilutions, with 91.2% of results at the mode (16 μg/ml) and 100% within 1 doubling dilution of the mode. Overall, there was a trend toward higher MIC results for both QC strains with the use of medium lots 2 and 3. These media generated four out-of-range MIC results reported by three different laboratories.

All vancomycin MIC results against the QC strains tested (not shown) were within published QC ranges (100% overall), and thereby provided a valid internal control for the study.

QC ranges for exebacase were calculated using both CLSI M23-A3 criteria and RangeFinder methods, which resulted in the recommendation of a 4-log_2_-unit QC range for both ATCC strains. Both S. aureus ATCC 29213 and E. faecalis ATCC 29212 QC ranges had a bimodal distribution of values, and no statistical differences as determined by the RangeFinder statistical program analysis were observed. The ranges recommended to the CLSI Subcommittee for Antimicrobial Susceptibility Testing were 0.25 to 1 μg/ml for S. aureus ATCC 29213 and 8 to 64 μg/ml for E. faecalis ATCC 29212. Importantly, none of the eight participating labs noted a trailing effect when testing the two QC strains.

## DISCUSSION

The results of this multilaboratory study demonstrated that the new exebacase broth microdilution MIC method developed by ContraFect using CAMHB supplemented with 25% horse serum and 0.5 mM DTT produced acceptable intralaboratory and interlaboratory reproducibility of MIC results with replicate testing of CLSI QC reference strains S. aureus ATCC 29213 and E. faecalis ATCC 29212. The results confirmed that MIC endpoints determined for both of the two ATCC strains tested were easy to read by different laboratory personnel and generated reproducible results. The studies described here met CLSI document M23-A3 (6) requirements for tier 2 QC studies to establish MIC ranges for this novel protein antibacterial agent.

**FIG 1:**
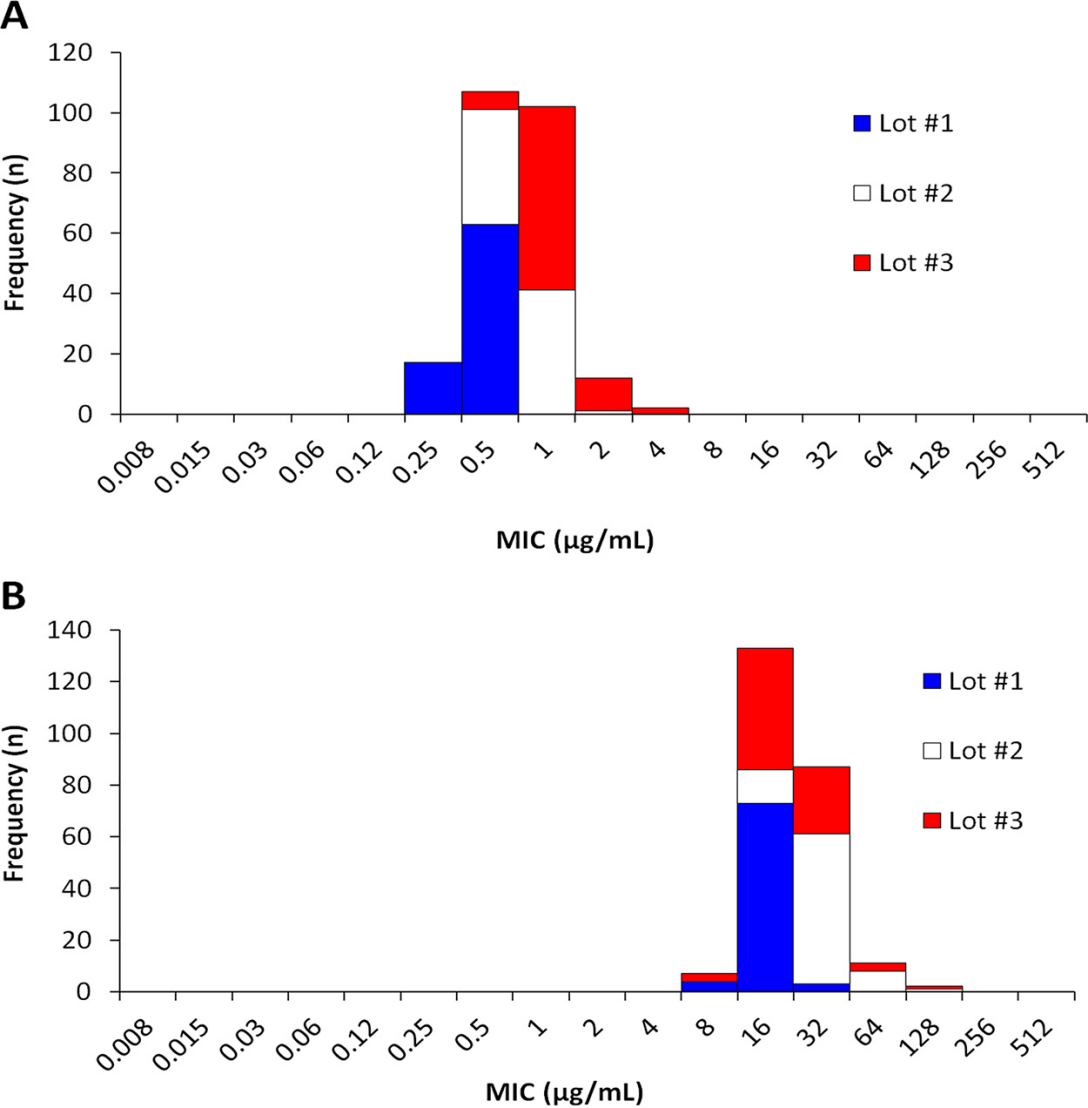
Distribution of exebacase broth microdilution MIC test results against ATCC QC strains S. aureus ATCC 29213 (A) and E. faecalis ATCC 29212 (B). The medium lots are indicated as follows: blue, medium lot 1; white, medium lot 2; red, medium lot 3.

The MIC data generated by the eight participating laboratories produced a four-dilution QC range for each of the QC strains tested, S. aureus ATCC 29213 and E. faecalis ATCC 29212 (Fig. 1). Table 2 summarizes the MIC QC ranges evaluated in this study and approved by the CLSI AST Subcommittee to be used with the exebacase broth microdilution method. For both of the ATCC strains evaluated, 99.2% of MIC values reported by the eight laboratories were within range. Two different laboratories (lab 2 and 3) each reported a single out-of-range exebacase MIC result of 4 μg/ml against S. aureus ATCC 29213. Various factors such as growth medium, pH, divalent cation concentration, and age of medium are known to influence broth microdilution susceptibility test results (14). Out-of-range QC results may be directly attributed to the medium used for testing. In this study, the commercial source of CAMHB influenced the extent of MIC variability, with medium lots that require additional cation supplementation having 3 and 4 log_2_ dilutions against S. aureus ATCC 29213 and 4 and 5 log_2_ dilutions against E. faecalis ATCC 29212. This contrasts with medium lot 1, where the MIC range was 2 and 3 log_2_ dilutions against ATCC 29213 and ATCC 29212, respectively (Table 1).

**TABLE 2:** Summary of CLSI-approved broth microdilution MIC QC ranges for exebacase

Strain	MIC range (μg/ml)	Occurrences (%) in range
S. aureus ATCC 29213	0.25–2	99.2
E. faecalis ATCC 29212	8–64	99.2

In conclusion, the results and analyses from this study led to the approval of exebacase MIC QC ranges summarized in Table 2 by the CLSI Subcommittee for Antimicrobial Susceptibility Testing and subsequent publication in CLSI standard M100 (15). Exebacase is the first protein-based antibacterial biologic to be reviewed by a standards development organization and to establish QC ranges for a broth microdilution method. The approved QC ranges will allow continued monitoring of QC data to ensure that exebacase MIC results generated by a variety of laboratories during clinical development can reliably assess the accuracy and reproducibility of MIC results and test performance when testing clinical isolates. A superiority design phase 3 study to assess the efficacy and safety of exebacase in patients with S. aureus bacteremia, including right-sided endocarditis is ongoing (https://clinicaltrials.gov/ct2/results?cond=&term=NCT04160468&cntry=&state=&city=&dist=). Continued monitoring of QC data during testing of clinical trial and surveillance isolates will allow further assessment of expected exebacase QC ranges to establish whether the current QC ranges published in CLSI document M100 should remain unchanged or require modification. As susceptibility testing of additional Staphylococcus species is performed, reading of MIC endpoints, MIC reproducibility, and interlaboratory variation will be further assessed and consideration given to recommendations for specific instructions for reading and interpreting MIC tests (e.g., 80% or 100% inhibition), including photographs to assist the reader with these instructions.
